# Carbon Nanofiber-Reinforced Carbon Black Support for Enhancing the Durability of Catalysts Used in Proton Exchange Membrane Fuel Cells Against Carbon Corrosion

**DOI:** 10.3390/membranes15010003

**Published:** 2024-12-26

**Authors:** Minki Sung, Hyeonseok Yi, Jimin Han, Jong Beom Lee, Seong-Ho Yoon, Joo-Il Park

**Affiliations:** 1 Interdisciplinary Graduate School of Engineering Sciences, Kyushu University, 6-1 Kasuga-koen, Kasuga, Fukuoka 816-8580, Japan; mksung@vina.co.kr; 2 Fuel-Cell Division Building 1, 253 Cheomdansaneop 1-ro, Bongdong-eup, Wanju-gun 55313, Republic of Korea; jmhan@vina.co.kr; 3 Carbon Materials Research Group, Research Institute of industrial Science & Technology (RIST), Pohang 37673, Republic of Korea; h.yi@rist.re.kr; 4 Department of Chemical & Biological Engineering, Hanbat National University, Daejeon 34158, Republic of Korea; whdqja0020@naver.com; 5 Institute for Materials Chemistry and Engineering, Kyushu University, 6-1 Kasuga-koen, Kasuga, Fukuoka 816-8580, Japan

**Keywords:** proton exchange membrane fuel cell, membrane electrode assembly, carbon nanofiber, carbon corrosion, hybrid support

## Abstract

This study addresses the critical challenge of carbon corrosion in proton exchange membrane fuel cells (PEMFCs) by developing hybrid supports that combine the high surface area of carbon black (CB) with the superior crystallinity and graphitic structure of carbon nanofibers (CNFs). Two commercially available CB samples were physically activated and composited with two types of CNFs synthesized via chemical vapor deposition using different carbon sources. The structure, morphology, and crystallinity of the resulting CNF–CB hybrid supports were characterized, and the performances of these hybrid supports in mitigating carbon corrosion and enhancing the PEMFC performance was evaluated through full-cell testing in collaboration with a membrane electrode assembly (MEA) manufacturer (VinaTech, Seoul, Republic, of Korea), adhering to industry-standard fabrication and evaluation procedures. Accelerated stress tests following the US Department of Energy protocols revealed that incorporating CNFs enhanced the durability of the CB-based hybrid supports without compromising their performance. The improved performance of the MEAs with the hybrid carbon support is attributed to the ability of the CNF to act as a structural backbone, facilitate water removal, and provide abundant edge plane sites for anchoring the platinum catalyst, which promoted the oxygen reduction reaction and improved catalyst utilization. The findings of this study highlight the potential of CNF-reinforced CB supports for enhancing the durability and performance of PEMFCs.

## 1. Introduction

Driven by the pressing need to curtail reliance on fossil fuels and mitigate the detrimental effects of greenhouse gas emissions, the global pursuit for sustainable energy solutions has intensified in recent years. Fuel cells have emerged as a promising solution in this endeavor, offering a clean and efficient pathway for the direct conversion of chemical energy into electricity [1,2,3,4,5,6]. Among the diverse array of fuel cell technologies, proton exchange membrane fuel cells (PEMFCs) have garnered significant attention owing to their unique attributes, such as their low operating temperature, high power density, and inherent scalability that make them particularly well suited for various applications, ranging from transportation and stationary power generation to portable electronic devices [7,8,9,10,11,12,13,14].

Despite their promise, PEMFCs face a critical challenge that hinders their widespread deployment, which is the degradation of the carbon support within the cathode catalyst layer [15,16,17,18,19]. The carbon support used in PEMFCs, typically composed of porous materials such as carbon black (CB), plays a vital role in maximizing the active surface area of platinum-based catalysts and facilitating the oxygen reduction reaction at the cathode [20,21]. However, under the harsh operating conditions of the PEMFC, the carbon support is susceptible to corrosion, which can significantly impair the performance and longevity of the fuel cell [22,23,24]. PEMFCs consist of some core parts, such as gas diffusion layer (GDL), membrane electrode assembly (MEA), and bipolar plates. Of them, MEA is the key component of PEMFCs, where the electrochemical reactions that generate electricity occur. It is named as such because it comprises a proton exchange membrane sandwiched between two electrodes, forming a cohesive assembly. The MEA’s structure facilitates the transport of reactants (hydrogen and oxygen) to the catalyst sites while also conducting protons and electrons to complete the electrical circuit.

The corrosion of carbon supports in PEMFC cathodes is a complex phenomenon, primarily driven by the electrochemical oxidation of carbon in the presence of water and oxygen. This oxidation process, exacerbated by low electrode potentials and frequent start-up/shutdown cycles, leads to the formation of surface oxides that subsequently react with water to produce carbon dioxide. The loss of carbon atoms not only decreases the active surface area of the loaded catalyst but also compromises the structural integrity of the electrode, resulting in catalyst detachment and agglomeration. During fuel cell operation, the platinum catalyst undergoes degradation, exhibiting an increase in particle size, a decrease in surface area, and a reduction in electrochemical active surface area (ECSA). This degradation occurs through two primary mechanisms: Ostwald ripening and particle agglomeration. Ostwald ripening involves the dissolution of smaller particles and their redeposition onto larger particles, while particle agglomeration stems from the detachment and subsequent aggregation of particles due to carbon support corrosion.

Platinum catalyst degradation can be categorized into several modes, including carbon support corrosion, dissolution of active metal components, detachment of catalyst nanoparticles, electrochemical Ostwald ripening, and nanoparticle coalescence. Carbon corrosion typically occurs during start-up/shutdown or hydrogen starvation events, where the cathode potential can reach up to 1.5V (vs NHE). For platinum-based alloy catalysts, the dissolution of reactive elements, leading to dealloying, is a major contributor to performance degradation. Even the relatively stable platinum is not entirely immune to dissolution under harsh corrosive environments. Nanoparticle sintering arises from electrochemical Ostwald ripening and nanoparticle coalescence. Electrochemical Ostwald ripening can be attributed to the chemical potential difference between nanoparticles of different sizes. Essentially, smaller nanoparticles tend to dissolve into the electrolyte and redeposit onto larger nanoparticles. Consequently, smaller nanoparticles disappear, and larger nanoparticles grow in size, resulting in ECSA loss. Nanoparticle coalescence occurs when nanoparticles are not firmly anchored to the matrix and readily agglomerate into larger particles, increasing the number of inaccessible platinum atoms in the inner core and leading to significant ECSA loss. Carbon corrosion accelerates these degradation processes, ultimately impacting the lifespan of the fuel cell [25,26].

These detrimental effects ultimately manifest as a decline in fuel cell performance, reduced efficiency, and shortened lifespan. Consequently, various support materials are being developed to minimize carbon corrosion while maximizing the electrochemical activity of the electrode [27,28]. Potential alternative materials include highly crystalline carbon materials such as graphite, carbon nanotubes (CNTs), and carbon nanofibers (CNFs), which exhibit high crystallinity and chemical stability, leading to superior resistance to carbon corrosion. Metal oxides like titanium oxide, tin oxide, and tungsten oxide also hold promise due to their high corrosion resistance and electrical conductivity, making them suitable candidates for replacing carbon supports. Additionally, conductive polymers, such as polyaniline and polypyrrole, offer the possibility of supporting catalysts without a carbon support due to their inherent electrical conductivity and catalytic activity [28].

Among these alternatives, highly crystalline carbon supports have been extensively studied for their ability to mitigate carbon corrosion. For PEMFC applications, it is crucial to develop new carbon support materials that facilitate uniform platinum dispersion, exhibit strong resistance to electrochemical corrosion, and possess high electrical conductivity. Many researchers are focusing on new carbon supports with unique structures and characteristics that are believed to enhance the performance and lifespan of PEMFCs. Furthermore, there are reports indicating that the optimization of catalyst performance can be achieved by employing fibrous carbon nanotubes and carbon nanofibers [29]. The most commonly accepted approach is to increase the degree of graphitization to improve resistance to carbon corrosion. The unique surface structure and excellent mechanical and thermal properties of CNTs and CNFs are expected to offer significant potential for catalyst supports [30]. While carbon corrosion directly impacts the decrease in the activity of electrode catalysts, from the perspective of the membrane electrode assembly (MEA), the issue of oxygen diffusion in the catalyst layer caused by the oxidation of the carbon support leads to more severe cell performance degradation [31].

Along with these research trends in crystalline carbon supports and inspired by previous studies [32,33], to overcome the aforementioned challenges, we planned to develop a novel hybrid support that harnesses the synergistic properties of two distinct carbon materials: CB with a high surface area and carbon nanofibers (CNFs) with superior crystallinity and a graphitic structure, imparting enhanced corrosion resistance to the support. Our particular focus was to investigate whether the incorporation of a certain amount of CNFs alone could provide enhanced durability against carbon corrosion. By judiciously combining these two materials, we aimed to create a robust support that retains the high surface area essential for catalyst dispersion while fortifying the electrode against carbon corrosion. In addition to improving durability against carbon corrosion, several studies have reported enhanced electrochemical activity when using CNFs or CNTs alone or in composites. This enhancement is thought to stem from the formation of a conductive network by the fibrous nanocarbon materials between the CB particles [34,35,36].

To synthesize CNFs with tailored morphology and properties, we employed a metal catalyst assisted chemical vapor deposition (CVD) method to produce two types of CNFs with different morphologies using different carbon sources. The CVD-synthesized CNFs were then meticulously integrated with activated CB to fabricate CNF-CB hybrid supports. The efficacy of these CNF-CB hybrid supports in mitigating carbon corrosion and enhancing the PEMFC performance was rigorously evaluated using a comprehensive suite of electrochemical measurements and accelerated stress tests (ASTs) conducted in accordance with the US Department of Energy (DOE) protocols. Our findings highlight the potential of these novel supports to substantially improve the durability and performance of the PEMFCs, paving the way for their broader integration into sustainable energy futures.

## 2. Materials and Methods

### 2.1. Sample Preparation

Two CB samples, Hexing 50%C and Denka Li-435, designated as CBA and CBB, respectively, were used as starting materials for preparing the hybrid support. The two CB samples were physically activated by first heating them to 930 °C at 10 °C/min rate under nitrogen flow at 100 mL/min flow rate, and then introducing steam as an activating agent at 400 mL/min flow rate, along with nitrogen as the carrier gas at 100 mL/min. The activation process was conducted for 4.5 h, and the activated CBA and CBB samples are denoted as CBAa and CBBa, respectively. Figure S2 showing that the inorganic ion species are not exist on CBA after activation.

CNFs were synthesized via chemical vapor deposition (CVD). First, a metal catalyst with a Fe:Ni:Co:Mg weight ratio of 60:10:10:20 was prepared via the sol-gel method. For this, metal nitrate precursors were mixed with citric acid at a molar ratio of 1:0.625 and heated to 250 °C. The resulting mixture was subsequently oxidized at 350 °C to obtain the mixed metal catalyst.

CNFs were synthesized using 1 g of the prepared mixed metal catalyst under a CO gas flow of 160 mL/min and H_2_ gas flow of 40 mL/min. Platelet-type CNFs were synthesized at 610, 620, and 630 °C for 4 h. Herringbone-type CNFs were synthesized using an ethylene (C_2_H_4_) gas flow of 100 mL/min and H_2_ flow of 100 mL/min at the same temperatures (610, 620, and 630 °C) for 1 h. The yields of the platelet-type and herringbone-type CNFs were 62-fold and 30-fold, respectively. After the CNF synthesis, the metal catalyst was removed using hydrochloric acid.

The selected CNFs were composited with CBAa and CBBa at CNF:CB weight ratios of 5:95 and 10:90 by ball milling the mixtures in a ball mill using 3 mm zirconia balls for 24 h. The final hybrid samples are designated as CBXa_YY, where X represents the type of activated CB (A or B) and YY represents the weight percentage of the CNF.

### 2.2. Material Characterization

To obtain pore structural information, the N_2_ adsorption–desorption isotherms at 77K of the hybrid supports were obtained using a surface area analyzer (Nova-e series; Anton Paar QuantaTec Inc., Boynton Beach, FL, USA). Pore structural information, including the specific surface area and total pore volume, was analyzed using the quenched solid density functional theory (QSDFT) method [37,38]. The mesopore ratio was calculated as follows:

Mesopore ratio [%] = (volume of 2–50 nm mesopores/total pore volume) × 10(1)

The morphological characteristics of the samples were investigated using scanning electron microscopy (SEM; EM-30N; COXEM, Daejeon, Republic of Korea) and Cs-corrected transmission electron microscopy (TEM; JEM-ARM200F; JEOL, Tokyo, Japan). The d_002_ and Lc(002) values were determined through X-ray diffraction (XRD) analysis (Mini-flex600; Rigaku Corp., Tokyo, Japan) to assess the anticorrosion factor of the MEA.

The degree of graphitization of the carbon support samples was evaluated by analyzing the interlayer spacing (d002) and the stacking height (Lc(002)) obtained from X-ray diffraction (XRD) patterns. The interlayer spacing was calculated using Bragg’s law with Cu Kα radiation (λ = 1.5418 Å) and the measured Bragg angle (θ) from the XRD patterns. The stacking height was estimated using the Scherrer equation, where β002 is the full width at half maximum (FWHM) of the (002) diffraction peak in radians. These parameters were calculated using the following equations:

d002 = λ/(2 × sin θ)(2)

Lc(002) = K × λ/(β002 × cos θ)(3)

### 2.3. Fuel Cell Evaluation

To adhere to industry-standard MEA fabrication and evaluation methods, this study was conducted in collaboration with VinaTech, an MEA manufacturer. For the full-cell evaluation, electrodes were fabricated using the synthesized 50 wt% Pt/C catalyst on an active area of 25 cm^2^, adhering to VinaTech’s standard protocol (TEM image of Pt/C is shown in Figure S4). For MEA fabrication, a catalyst slurry was prepared as follows: 1 g of 50 wt% Pt/C catalyst and 2 g of ionomer were dispersed in a mixture of 2 g of deionized (DI) water and 2 g of isopropyl alcohol (IPA) to achieve a 1:2 mass ratio of carbon to ionomer. The mixture was thoroughly mixed until a homogeneous slurry was formed. The platinum loading of all electrodes was adjusted to 0.3 mgPt/cm^2^ using a handheld X-ray fluorescence (XRF) gun (Handheld XRF VANTA series, YOUNG IN AT). Subsequently, seven-layer MEAs were manufactured by assembling the catalyst coated membrane (CCM) with additional carbon paper gas diffusion layers (GDLs) and sub gaskets, following VinaTech’s decal method and internal processes (The cross-section SEM image of MEA is shown in Figure S1). Subsequently, seven-layer MEAs were manufactured according to the internal processes of VinaTech. Prior to evaluation, an electrode activation process was performed to enhance MEA performance. Polarization curves were measured to evaluate the performance of MEAs. The measurements were carried out at a cell temperature of 60 °C, 100% RH, and ambient pressure. H_2_ and air were used as the anode and cathode gases, respectively, with a stoichiometry of 1.5:2. The current range was set from 0 to 50 A.

Thereafter, fuel cell performance evaluation and accelerated stress tests (ASTs) were conducted in accordance with the US DOE protocols [39]. Durability testing was performed according to the support AST protocol outlined in the DOE. The support AST was conducted using a triangle sweep cycle between 0.1 and 1.5 V at a scan rate of 0.5 mV/s. The temperature of both the electrodes (anode and cathode) was maintained at 80 °C, and fuel was supplied at 100% relative humidity (RH) and 80 °C. Hydrogen (H_2_) at 200 sccm and nitrogen (N_2_) at 75 sccm were used as the anode and cathode gases, respectively. The test was performed for a total of 5000 cycles (cycle time: 6 s). Electrochemical measurements were performed before and after the support AST using a potentiostat (VSP; BioLogic, Seyssinet-Pariset, France).

## 3. Results and Discussion

### 3.1. Properties and Selection of CNFs

**Figure 1:**
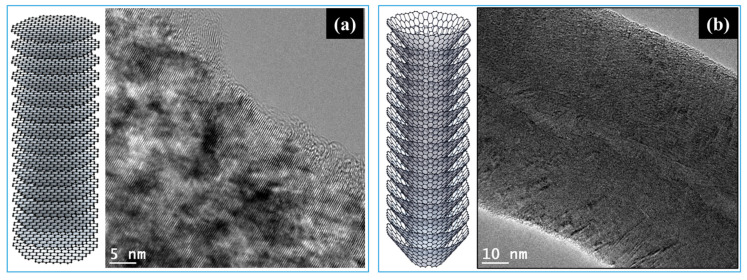
Transmission electron microscopy images and schematic illustration of the carbon structures in (**a**) platelet-type and (**b**) herringbone-type carbon nanofibers.

Table 1 presents the properties of the six types of carbon nanofibers (CNFs) synthesized via chemical vapor deposition (CVD), including their yield, specific surface area, fiber diameter, and degree of crystallinity. The CNFs were classified as either platelet- or herringbone-type based on the arrangement of their graphitic layers, as observed by transmission electron microscopy (TEM). Figure 1 illustrates the distinct structural differences between these two types of CNFs. The platelet-type CNFs exhibit a planar structure with horizontally arranged graphitic layers (graphene sheets), while the herringbone-type CNFs possess a characteristic structure with stacked and rolled graphitic layers that appear diagonally arranged in the TEM images.

The arrangement of graphitic layers in CNFs has significant implications for their application as catalyst supports in proton-exchange membrane fuel cells (PEMFCs). In contrast to carbon nanotubes (CNTs), in which the carbon surface consists predominantly of basal planes with only the tube ends exposing the edge planes [40], CNFs offer a greater extent of edge plane exposure. This distinction is crucial because, despite the superior electrical conductivity of CNTs owing to their basal plane surfaces, electrochemical reactions primarily occur at the edge planes. As illustrated in Figure 1, both the platelet- and herringbone-type CNFs exhibit exposed edge planes, providing abundant sites for anchoring the platinum catalyst and thereby facilitating more efficient electrochemical reactions. Consequently, CNFs have a distinct advantage over CNTs as catalyst supports for electrochemical applications such as PEMFCs [40].

**Figure 2:**
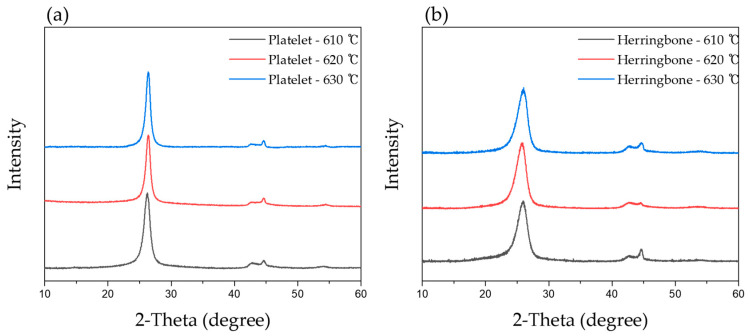
X-ray diffraction spectra of carbon peaks. (**a**) Platelet-types and (**b**) herringbone-types.

Figure 2 shows the X-ray diffraction spectra of carbon peaks for the fabricated CNFs. In this analysis, the (002) peak of carbon, which corresponds to the stacking of graphitic layers, is clearly observed. The platelet-type CNFs (Figure 2a) exhibit a sharper and narrower full width at half maximum (FWHM) for the (002) peak compared to the herringbone-type CNFs, indicating a higher degree of crystallinity. This observation is consistent with the TEM analysis, which revealed a more ordered arrangement of graphitic layers in the platelet-type CNFs. The higher crystallinity of platelet-type CNFs can be attributed to the preferential growth of graphitic layers along the fiber axis during the CVD synthesis process.

While the platelet-type CNFs synthesized using CO as the primary reactant gas tended to exhibit higher crystallinity, the herringbone-type CNFs prepared using ethylene as the carbon source were also of high crystallinity, as confirmed by XRD analysis. Moreover, although the platelet-type CNFs were obtained with approximately twice the yield of the herringbone-type CNFs, the overall yield and productivity of the herringbone-type CNFs were superior upon considering the hourly yield and higher cost of the CO gas compared with that of the ethylene gas.

**Figure 3:**
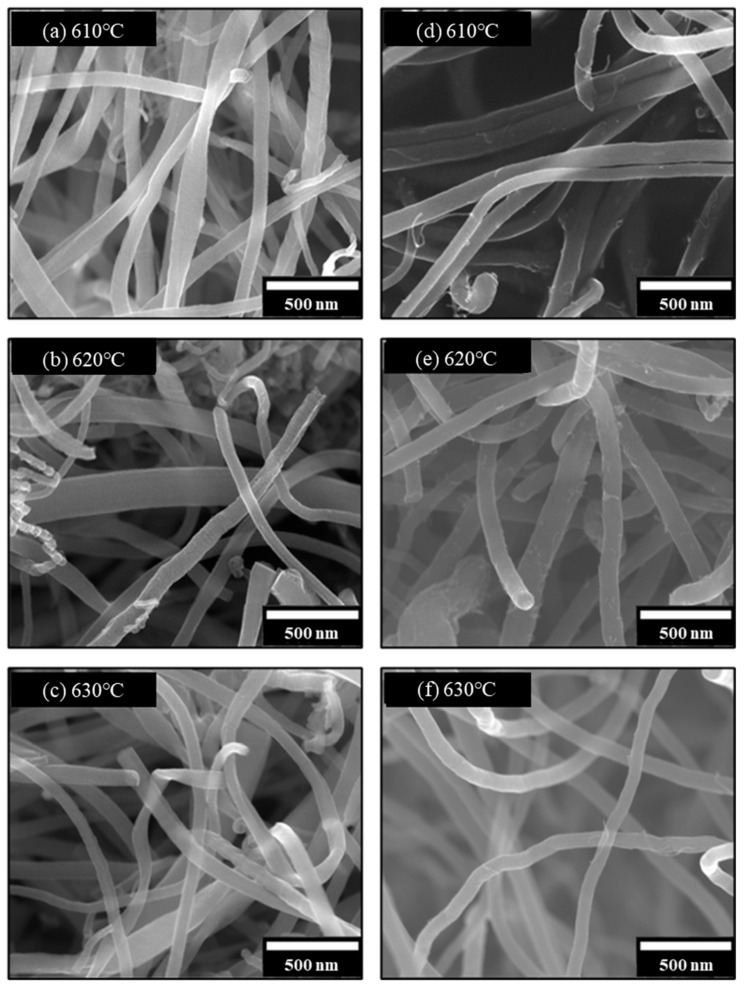
Scanning electron microscopy images of the carbon nanofibers (CNFs) synthesized at different temperatures using different carbon sources: (**a**–**c**) platelet-type and (**d**–**f**) herringbone-type CNFs.

The morphologies of the synthesized CNFs were characterized using SEM and TEM. Figure 3a–c presents the SEM images of the platelet-type CNFs synthesized at 610, 620, and 630 °C, respectively, which reveal the formation of straight CNFs with good linearity. However, all three samples exhibit irregularly grown CNFs with small diameters and twisted graphitic layers, as shown in Figure 3b. Additionally, platelet-type CNFs generally showed significant variations in the fiber diameter.

Figure 3d–f shows the SEM images of the herringbone-type CNFs synthesized at 610, 620, and 630 °C, respectively. Although the herringbone-type CNFs exhibited relatively uniform fiber diameters, The CNFs synthesized at 630 °C (Figure 3f) exhibited the most uniform fiber diameter and minimal pyrolytic carbon deposition, compared to those synthesized at 610 and 620 °C (Figure 3d,e). Considering the productivity, yield, morphology, and properties, the herringbone-type CNFs synthesized at 630 °C were selected for the fabrication of CNF–CB hybrid supports. In particular, we focused on the fact that the synthesis of herringbone-type CNFs can be achieved with a relatively short reaction time (1 h) compared to the platelet-type CNFs, which require a longer CVD reaction time (more than 4 h) to ensure the desired fiber morphology and yield.

### 3.2. Properties of the CNF–CB Hybrid Supports

Table 2 presents the structural parameters of the CNF–CB hybrid supports prepared by combining the herringbone-type CNFs synthesized at 630 °C with activated CB samples, with CBAa and CBBa in weight ratios of 5:95 and 10:90, respectively. As the CNF content increased, the specific surface area, mesopore volume, and total pore volume of the hybrid support decreased. However, XRD analysis revealed an improvement in the crystallinity of the composite, as indicated by an increase in the crystallite size (Lc) and a decrease in the interlayer spacing (d_002_). The decrease in the specific surface area and pore volume is attributed to the incorporation of CNFs with a lower specific surface area than that of CB. The observed improvement in crystallinity reflects a tradeoff relationship between the crystallinity and pore volume, whereby the addition of highly crystalline CNFs increases the thickness of the carbon crystallites (Lc) in the hybrid carbon composite, but reduces the interlayer spacing between the graphitic layers (d_002_).

**Figure 4:**
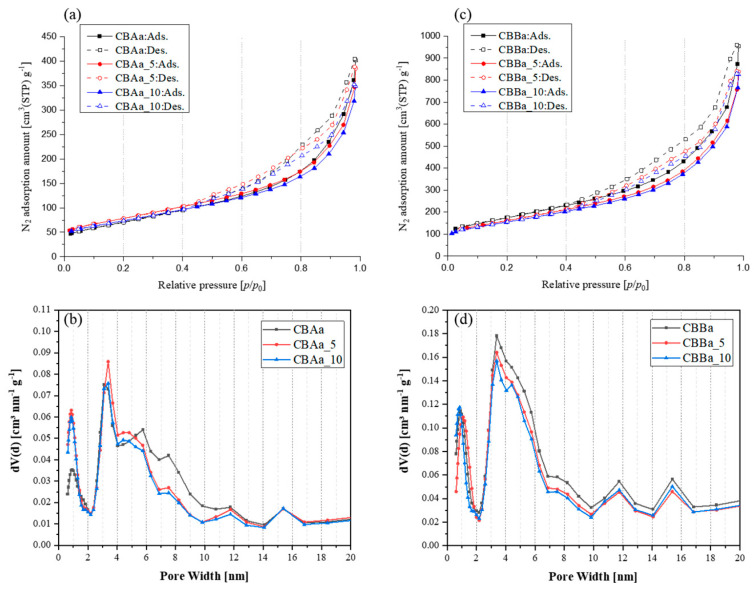
N_2_ adsorption–desorption isotherms recorded at 77K for CNF-CB hybrid supports: (**a**) CBAa series and (**c**) CBBa series. QSDFT pore size distributions: (**b**) CBAa series and (**d**) CBBa series.

The theoretical analysis of the CNF–CB hybrid supports using QSDFT [30,31] (Figure 4) revealed a decrease in the specific surface area and a corresponding decrease in the N2 adsorption–desorption capacity of the CNF–CB hybrid at 77 K with increasing CNF content. For the relatively low surface area CBAa sample (295.54 m^2^/g), the incorporation of CNFs led to a decrease in the mesopore volume in the 6 to 10 nm range and an increase in the micropore volume (Figure 4b). In contrast, for the higher surface area CBBa sample (628.59 m^2^/g), the addition of CNFs did not significantly affect the micropore volume, and both the specific surface area and pore volume decreased proportionally with the CNF content (Figure 4d).

**Figure 5:**
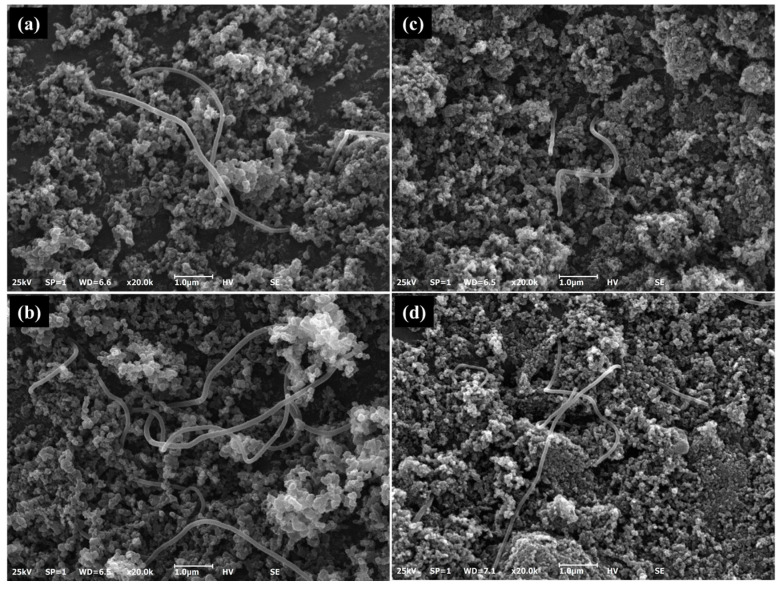
Scanning electron microscopy images of the CNF-CB hybrid supports: (**a**) CBAa_5, (**b**) CBAa_10, (**c**) CBBa_5, and (**d**) CBBa_10.

**Figure 6:**
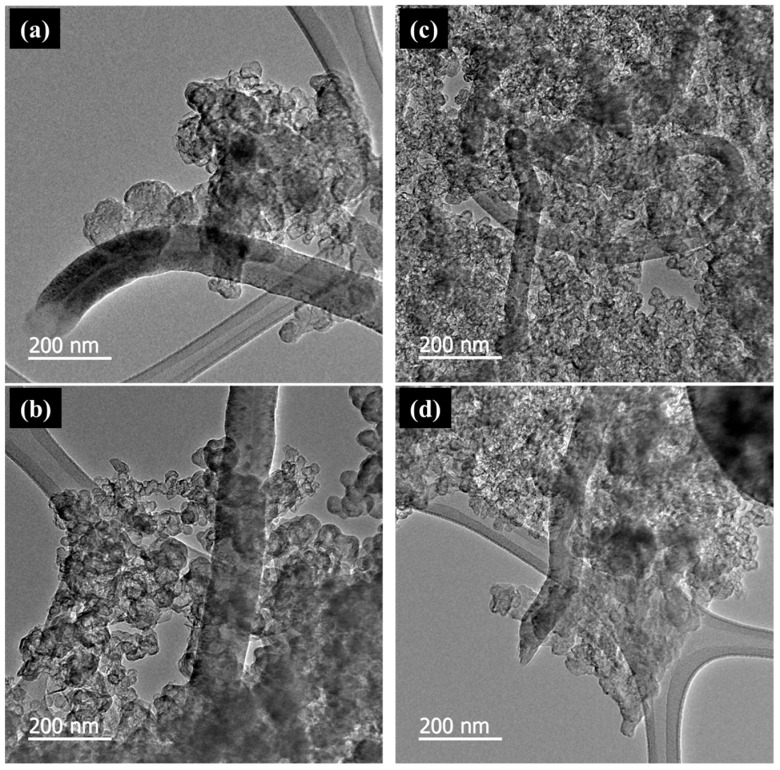
Transmission electron microscopy images of the CNF-CB hybrid supports: (**a**) CBAa_5, (**b**) CBAa_10, (**c**) CBBa_5, and (**d**) CBBa_10.

We confirmed from the SEM images (Figure 5) that CNF and CB were physically mixed properly by ball milling. As expected, the sample with 10 wt% CNF showed a higher CNF density than the sample with 5 wt% CNF. TEM images (Figure 6) showed that CNF and CB particles remained mixed even after sonication, indicating the adhesion force formed between the two carbon materials.

### 3.3. Fuel Cell Evaluation Results

**Figure 7:**
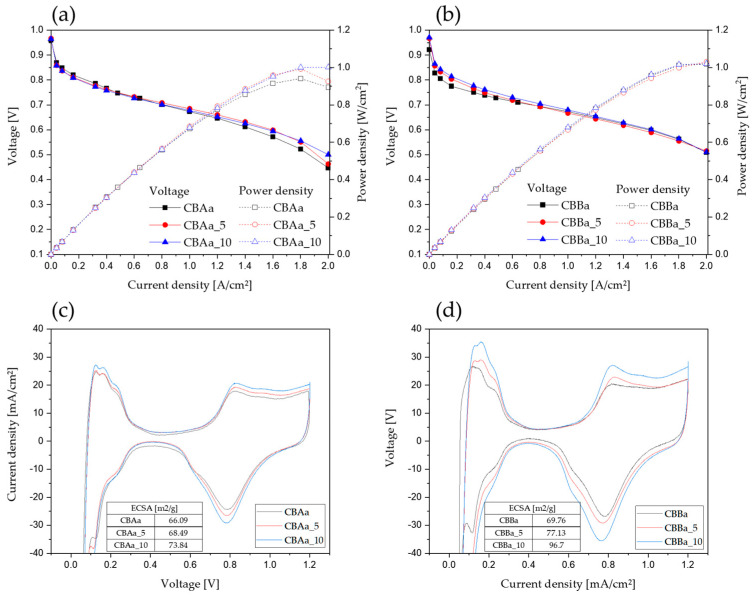
Initial performance evaluation and electrochemically active surface areas of MEAs with CNF-CB hybrid supports and reference samples. (**a**,**c**) CBAa series and (**b**,**d**) CBBa series.

The two activated commercial CB samples, CBAa and CBBa, were used as references in the electrochemical performance and durability evaluations of the CNF–CB hybrid supports. Single-cell MEA performance evaluation was conducted in collaboration with VinaTech, a manufacturer of MEAs for PEMFCs, and the full cell test results are presented in Figure 7. All the MEAs were fabricated under identical conditions, except for the cathode catalyst, which was varied according to the specific sample. To ensure consistency and industry relevance, MEA fabrication and evaluation procedures adhered to the protocols established by VinaTech. The electrodes were prepared using a 50 wt% platinum catalyst loaded onto a 25 cm^2^ active area of the hybrid support. Seven-layer MEAs, incorporating gas diffusion layers and gaskets, were constructed according to the internal specifications of VinaTech. Before the performance evaluation, an activation procedure was performed to optimize the MEA performance. The fuel cell performance and ASTs of the catalyst support were measured in accordance with the U.S. DOE protocols. The degradation rate of the MEA was calculated using the voltage measured at 1.6 A/cm^2^ as follows:

Degradation rate = (Voltage after AST − Initial voltage)/(Initial voltage) × 100(4)

The initial performance results of the CBAa series of samples (CBAa, CBAa_5, and CBAa_10) are presented in Figure 7a and Table 3. Both CBAa_5 and CBAa_10 composited with CNFs showed a slightly improved performance than that of the pristine CBAa sample without the CNFs. Notably, CBAa_10 with a higher CNF content performed better than CBAa at 2.0 A/cm^2^. This can be attributed to the fibrous carbon network within the CBAa_10 based electrode. Flooding, which occurs when the water generated by the electrochemical reactions in the cathode is not efficiently removed, can hinder mass transport within the electrode, particularly at high current densities, leading to performance decline. The incorporation of CNFs in the support creates a fibrous network that facilitates water drainage from the cathode, enabling efficient mass transport and thus contributing to a relatively higher performance, even at high current densities [41]. For the CBBa samples, a slightly improved performance was observed in the low current density range (0–0.6 A/cm^2^) for the CBBa_5 and CBBa_10 samples, which were hybridized with CNFs. However, as shown in Figure 7b and Table 3, no significant difference was observed between the carbon supports with and without CNFs at higher current densities. In other words, the initial performance of the three samples, CBBa, CBBa_5, and CBBa_10, was not significantly affected by the CNF content.

Figure 7c,d show the electrochemical surface area (ECSA) of the CBAa and CBBa series with and without CNFs. The ECSA of the Pt/C catalyst was calculated by integrating the area under the hydrogen desorption peak in the cyclic voltammetry (CV) curve, specifically within the potential range of 0 V to 0.4 V. The current value at 0.4 V was used as the baseline for integration, and the area corresponding to current values above this baseline was integrated to determine the active surface area of the catalyst. The ECSA was then calculated as follows:

(5) $$\sum_{0V}^{0.4V}I\;(>I\;@0.4\;V)$$

The ECSA of CBBa (69.76 m^2^/g) was higher than that of CBAa (66.09 m^2^/g), which is attributed to the higher specific surface area of the CB in CBBa, resulting in better catalyst dispersion. As the CNF content increased, the ECSA of CBAa_5 and CBAa_10 increased to 68.49 m^2^/g and 73.84 m^2^/g, respectively (Figure 7c). Similarly, the ECSA of CBB_5 and CBB_10 increased to 77.13 m^2^/g and 96.7 m^2^/g, respectively (Figure 7d).

This result suggests that the fibrous morphology of CNFs contributed to forming a more robust electrode structure, which increased the active surface area of the catalyst. This observation is consistent with several reports that highlight the ability of fibrous carbon structures, such as CNFs, to form electrically conductive networks [36,37,38]. Although incorporating CNFs did not significantly affect the initial MEA performance, it led to a proportional increase in the ECSA with increasing CNF content.

**Figure 8:**
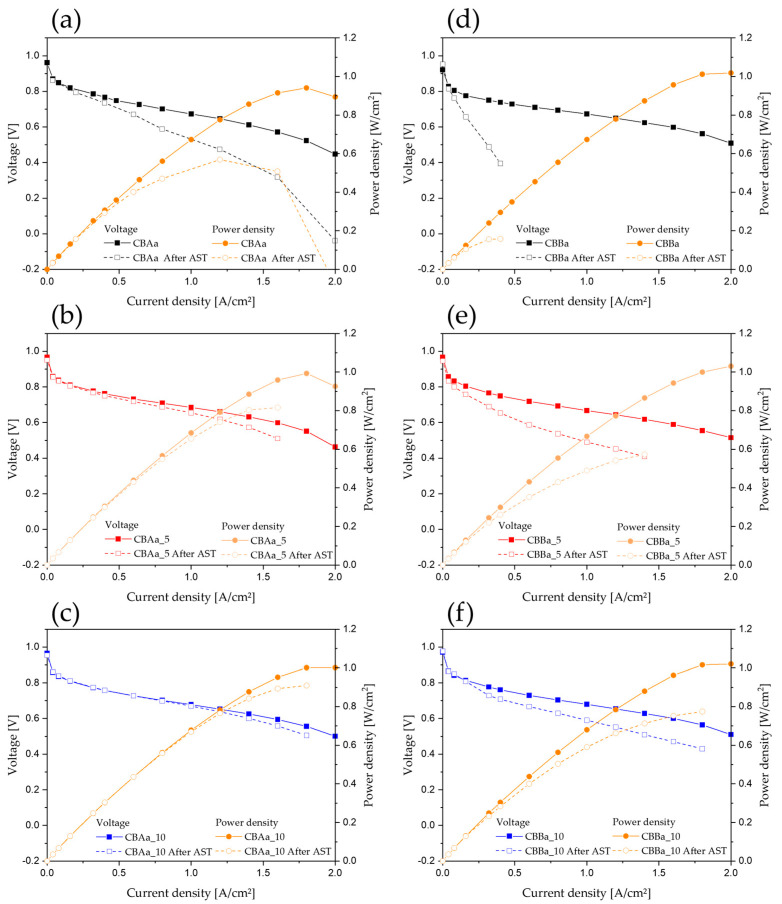
AST polarization curves and power density curves for full-cell MEAs: (**a**) CBAa, (**b**) CBAa_5, (**c**) CBAa_10, (**d**) CBBa, (**e**) CBBa_5, and (**f**) CBBa_10.

Figure 8 compares the performance of the different MEAs before and after the AST performed according to the DOE support durability protocol, which was designed to induce carbon corrosion. The corresponding power density curves are also shown. In certain cases, the carbon support was completely degraded, and further evaluation was not possible (in such cases, a hyphen (-) is included in the performance data in Table 3).

The CBAa samples exhibited a clear difference in durability depending on the CNF content; the degradation rate decreased with increasing CNF content. After the AST, the performance of CBAa and CBAa_5 declined significantly, and a very low voltage was observed at 1.6 A/cm^2^, which hindered further measurements. However, CBAa_10 retained high durability, exhibiting a degradation rate of 6.22% at 1.6 A/cm^2^ compared to its initial performance.

The CBBa samples exhibited a more rapid decline in durability after the AST than the CBAa samples. However, CNF–CBBa composites exhibited increased durability, and the trend of a progressive increase in durability with increasing CNF content was observed, as in the case of the CBAa series. The performance of CBBa and CBBa_5 degraded significantly after the AST, and a very low voltage was measured at 1.6 A/cm^2^, which made further measurements impossible. CBBa_10, the most durable sample in the CBBa series, exhibited a degradation rate of 21.80%, indicating its superior durability.

Although all CBBa series samples had higher ECSAs than the CBAa series samples, the CBAa series showed superior performance in terms of voltage, as observed in the polarization and power density curves. The more rapid decline in the durability of CBBa after the AST suggests that a higher ECSA may lead to the faster degradation of the platinum catalyst. Alternatively, it may indicate that carbon supports with higher specific surface areas are more susceptible to carbon corrosion at the cathode.

PEMFC MEA testing is sensitive to a wide range of experimental conditions and variables, including the materials used (GDL, membrane, electrode size and thickness, station type, catalyst loading and type, evaluation protocol, etc.) Therefore, we carefully controlled all experimental conditions, ensuring that the carbon support was the only variable, and observed the resulting performance and durability. These results suggest that a higher specific surface area and ECSA may not necessarily guarantee superior performance and durability. Carbon corrosion could potentially affect not only the carbon support and the platinum catalyst but also the entire MEA, ultimately leading to a significant reduction in lifespan. The polarization curves of CBAa and CBBa without CNFs (Figure 8a,d) indicate a complete degradation of the entire MEA under the harsh DOE support durability protocol. This study suggests the potential of CNF–CB composite supports, with their high crystallinity CNF component, to mitigate carbon corrosion. (Failed CNF–CB mixture sample is shown in Figure S3).

## 4. Conclusions

This study explored the development of hybrid supports by combining carbon nanofibers (CNFs) with commercially available carbon black (CB) to mitigate carbon corrosion in PEMFCs, a critical factor that can adversely affect their long-term performance and lifespan. These hybrid supports aimed to leverage the high surface area of CB for effective catalyst dispersion and the superior crystallinity and graphitic structure of CNFs for enhanced corrosion resistance.

Our study revealed that incorporating CNFs into the CB support could lead to several beneficial effects. The CNFs appeared to contribute to a more robust electrode structure, likely due to their fibrous morphology, which could facilitate the formation of a conductive network, promoting efficient charge transfer and catalyst utilization. The compositing of CNFs with CB seemingly mitigated the performance degradation caused by carbon corrosion. Accelerated stress tests demonstrated that incorporating CNFs significantly improved the durability of the hybrid supports.

The CBAa samples exhibited a difference in durability depending on the CNF content; the degradation rate decreased with increasing CNF content. The CBBa samples exhibited a more rapid decline in durability than the CBAa samples. However, CNF–CBBa composites exhibited increased durability with increasing CNF content. Although all CBBa series samples had higher ECSAs than the CBAa series samples, the CBAa series showed superior performance in terms of voltage. The more rapid decline in the durability of CBBa after the AST suggests that a higher ECSA may be associated with faster degradation of the platinum catalyst, or that carbon supports with higher specific surface areas are more susceptible to carbon corrosion.

These results suggest that a higher specific surface area and ECSA may not necessarily guarantee superior performance and durability. Carbon corrosion could potentially affect the entire MEA, leading to a significant reduction in lifespan. This study suggests the potential of CNF–CB composite supports to mitigate carbon corrosion.

This study explores the possibility of using CNF-reinforced CB supports to enhance the durability and performance of PEMFCs, and suggests a potential direction for the development of next-generation fuel cells.

## Figures and Tables

**Table 1:** CVD reaction conditions (gas, time, temperature), yield, and properties of platelet and herringbone-type carbon nanofibers.

Carbon Nano Fiber Type	CVD Reactant Gases (Total 200 mL/min)	Temp. (°C)	Time (Min)	Yield *^1^	Specific Surface Area (m^2^/g)	Diameter *^2^ (nm)	XRD	
							d_002_ (nm)	Lc(002) (nm)
Platelet	CO/H_2_ = 4/1 (Vol/Vol)	610	240	61.2	76	54–140	0.341	7.2
		620	240	62.2	104	53–214	0.339	10.1
		630	240	62.1	68	52–108	0.338	8.4
Herringbone	C_2_H_4_/H_2_ = 1/1 (Vol/Vol)	610	60	27.1	39	78–134	0.340	4.4
		620	60	35.0	38	43–125	0.340	4.8
		630	60	30.4	33	109–155	0.342	4.2

*^1^: The yield was calculated as the weight of CNFs produced divided by the amount of catalyst used. *^2^: The fiber diameter was determined by observing 100 CNFs and indicating the range.

**Table 2:** Pore structure/volume and crystallinity of the CNF–CB hybrid supports.

Sample	Pore Structural Parameters				XRD	
	Specific Surface Area (m^2^/g)	Mesopore Volume (cm^3^/g)	Total Pore Volume (cm^3^/g)	Mesopore Ratio (%)	Lc (nm)	d_002_ (nm)
CBAa	295.54	0.5437	0.5919	91.86	3.06	0.3544
CBAa_5	281.02	0.5231	0.5887	88.86	3.35	0.3541
CBAa_10	264.16	0.4869	0.5412	89.97	3.48	0.3500
CBBa	628.59	1.3750	1.4784	93.01	2.99	0.3532
CBBa_5	580.04	1.2129	1.2985	93.41	3.26	0.3501
CBBa_10	554.65	1.1883	1.2797	92.86	3.57	0.3488

**Table 3:** Accelerated stress test results for MEAs based on CNF–CB hybrid supports, following the DOE support durability protocol.

	V@0.16 A/cm^2^	V@0.4 A/cm^2^	V@1.0 A/cm^2^	V@1.6 A/cm^2^	Degradation Rate (%)
CBAa	0.820	0.766	0.673	0.572	Complete degradation
CBAa After AST	0.670	0.475	-	-	
CBAa_5	0.811	0.762	0.684	0.599	Complete degradation
CBAa_5 After AST	0.806	0.751	0.654	-	
CBAa_10	0.809	0.758	0.678	0.595	6.22
CBAa_10 After AST	0.812	0.759	0.670	0.558	
58CBBa	0.775	0.738	0.673	0.598	Complete degradation
CBBa After AST	0.657	0.395	-	-	
CBBa_5	0.804	0.749	0.667	0.589	Complete degradation
CBBa_5 After AST	0.758	0.653	0.490	-	
CBBa_10	0.814	0.761	0.680	0.601	21.80
CBBa_10 After AST	0.808	0.708	0.590	0.470	

## Data Availability

The data presented in this study are available on request from the corresponding author.

## Supplementary Materials

The following supporting information can be downloaded at: https://www.mdpi.com/article/10.3390/membranes15010003/s1, Figure S1: SEM images of MEA cross-sections; Figure S2: SEM-EDS analysis of CBAa; Figure S3: SEM images of a failed CNF-CB mixture sample; Figure S4: TEM image of platinum catalyst (Pt/C) supported on CNF-CB support.
