# Supplementary material for: Carbon Nanofiber-Reinforced Carbon Black Support for Enhancing the Durability of Catalysts Used in Proton Exchange Membrane Fuel Cells Against Carbon Corrosion
Source: Membranes (Basel). 2024 Dec 26;15(1):3. doi: 10.3390/membranes15010003 (PMC11767076; doi:10.3390/membranes15010003)
Supplement: Supplementary file 1 [file membranes-15-00003-s001.zip › membranes-3365094-supplementary.pdf]

Supplemental information

Carbon nanofiber-reinforced carbon black support for enhancing the durability of catalysts used in proton-exchange membrane fuel cells against carbon corrosion

Minki Sung [1,3], Hyeonseok Yi [2], Jimin Han [3], Jong Beom Lee[4], Seong-Ho Yoon [1,2,*], Joo-Il Park [4,*]

1 Interdisciplinary Graduate School of Engineering Sciences, Kyushu University, 6-1 Kasuga-koen, Kasuga, Fukuoka, 816-8580, Japan

2 Institute for Materials Chemistry and Engineering, Kyushu University, 6-1 Kasuga-koen, Kasuga, Fukuoka 816-8580, Japan

3 Fuel-cell division Building 1, 253 Cheomdansaneop 1-ro, Bongdong-eup, Wanju-gun, Jeonbuk-do 55313, Ko-rea

4 Department of Chemical & Biological Engineering, Hanbat National University, Daejeon 34158, Korea

5 Carbon Materials Research Group, Research Institute of industrial Science & Technology (RIST), Pohang 37673, Korea

* Correspondence: yoon@cm.kyushu-u.ac.jp (S.-H.Y), jipark94@hanbat.ac.kr (J.-I.P) ; Tel.: 81-92-583-7959 (S.-H.Y), 82-42-821-1530 (J.-I.P)

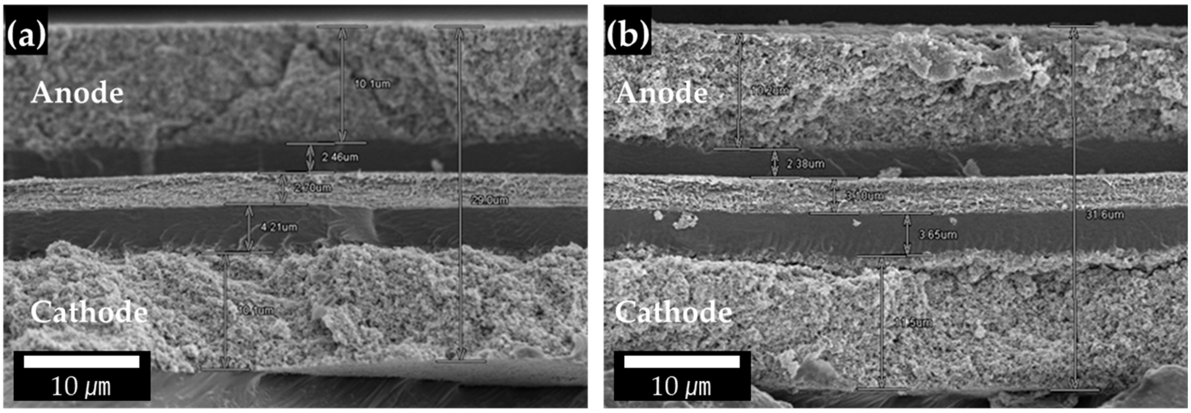

Figure S1. SEM images of MEA cross-sections: (a) CBAa and (b) CBAa_10

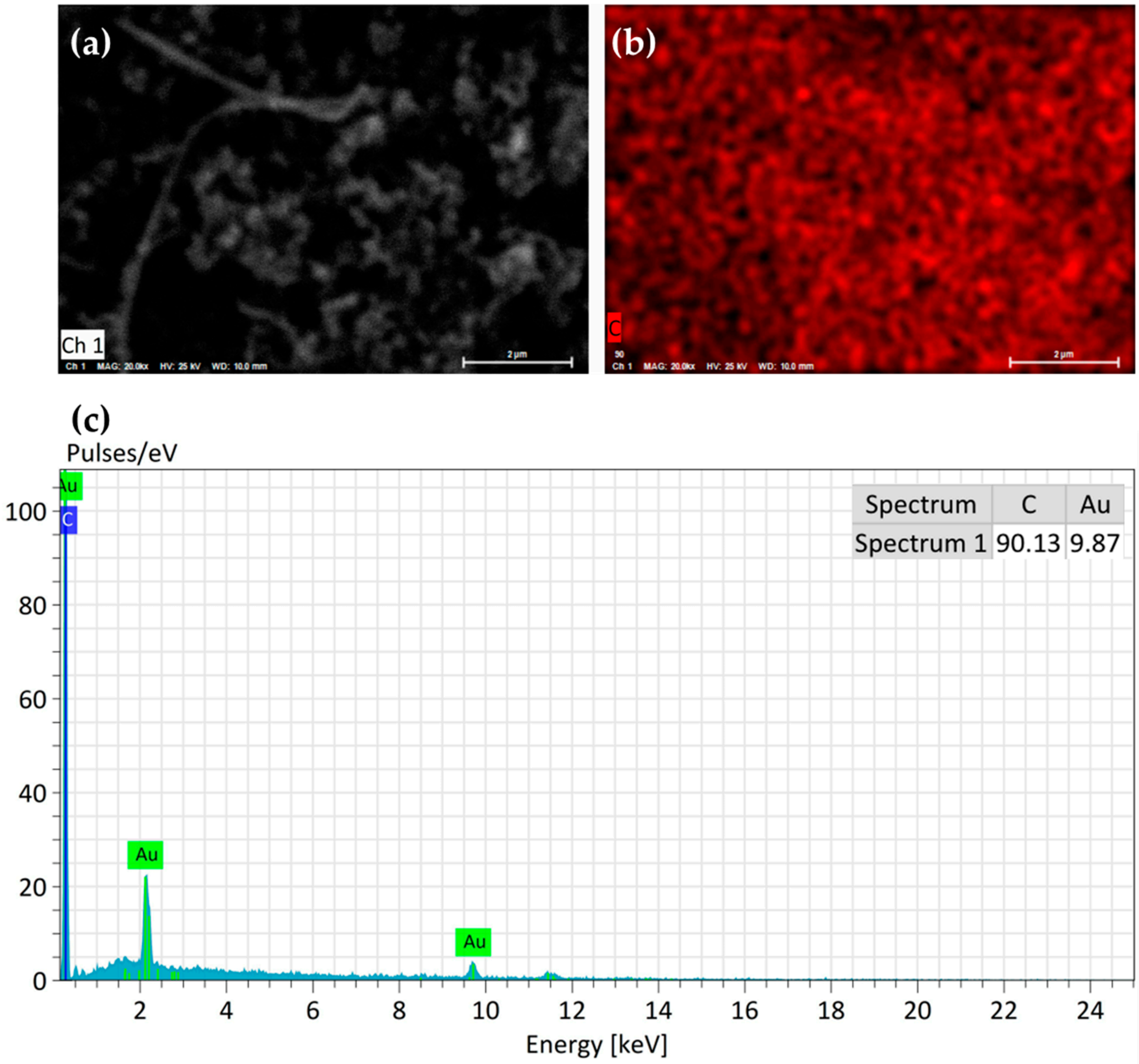

Figure S2. SEM-EDS analysis of CBAa: (a) SEM image at 20,000x magnification, (b) carbon mapping, and (c) EDS spectrum.

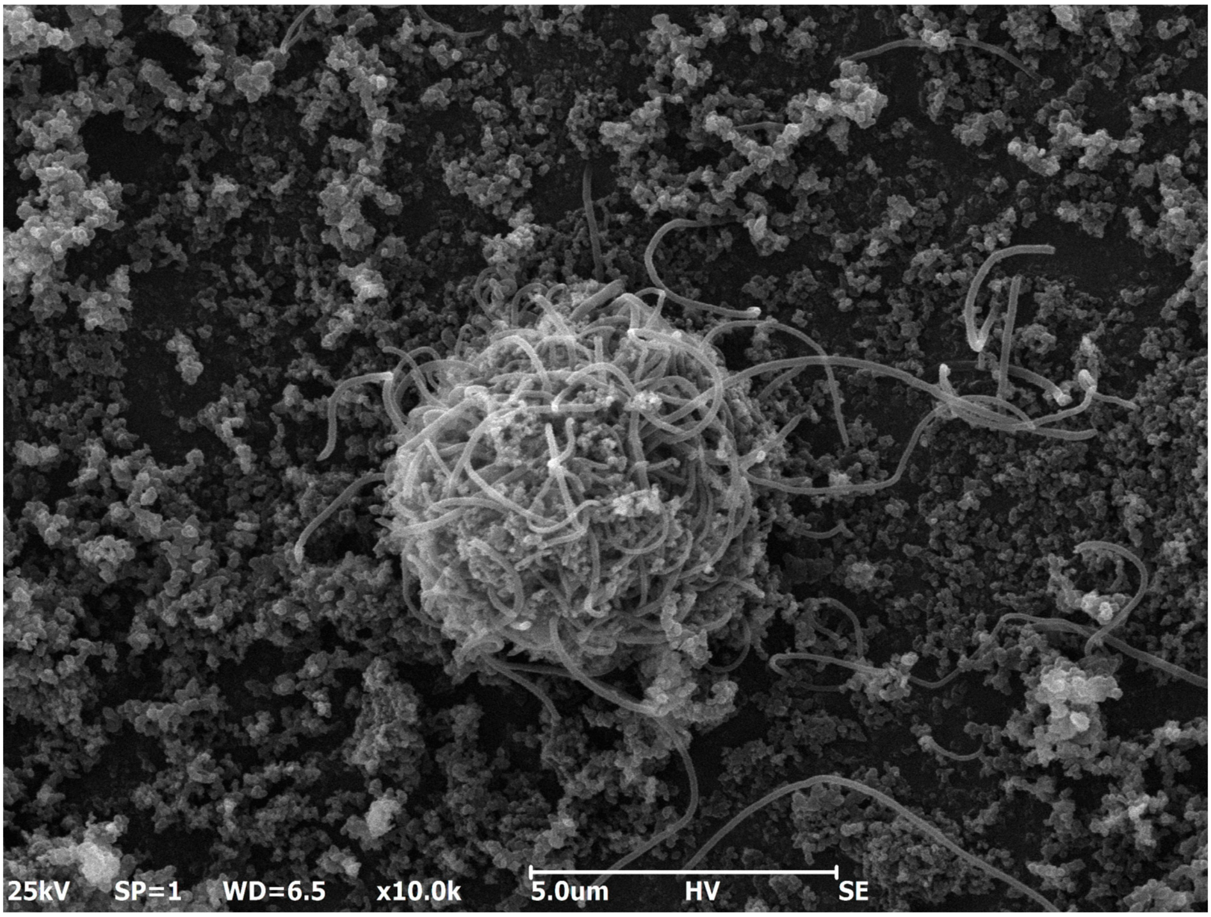

Figure S3. SEM image of a failed CNF-CB mixture sample

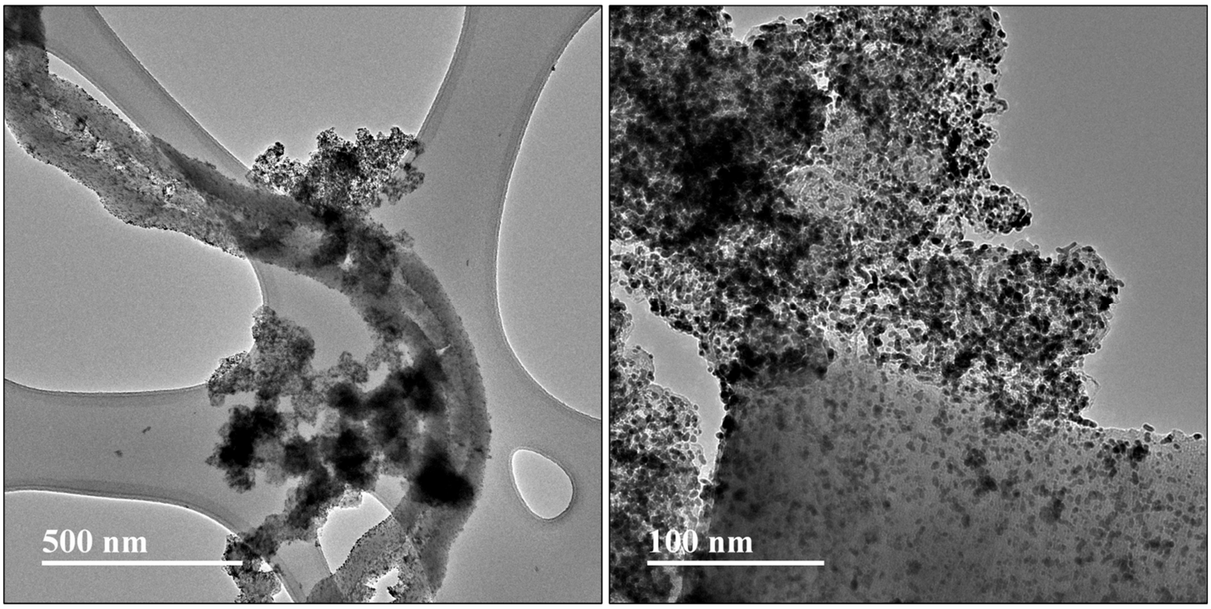

Figure S4. TEM image of platinum catalyst (Pt/C) supported on CNF-CB support
